# Prognostic models in coronary artery disease: Cox and network approaches

**DOI:** 10.1098/rsos.140270

**Published:** 2015-02-11

**Authors:** Antonio Mora, Rosa Sicari, Lauro Cortigiani, Clara Carpeggiani, Eugenio Picano, Enrico Capobianco

**Affiliations:** 1 Institute of Clinical Physiology, National Research Council, Pisa, Italy; 2 Laboratory of Integrative Systems Medicine (LISM), Institute of Clinical Physiology, National Research Council, Pisa, Italy; 3 Cardiology Division, Campo di Marte Hospital, Lucca, Italy; 4 Center for Computational Science, University of Miami, Coral Gables, FL 33146, USA

**Keywords:** coronary artery disease, cox models, mutual information network

## Abstract

Predictive assessment of the risk of developing cardiovascular diseases is usually provided by computational approaches centred on Cox models. The complex interdependence structure underlying clinical data patterns can limit the performance of Cox analysis and complicate the interpretation of results, thus calling for complementary and integrative methods. Prognostic models are proposed for studying the risk associated with patients with known or suspected coronary artery disease (CAD) undergoing vasodilator stress echocardiography, an established technique for CAD detection and prognostication. In order to complement standard Cox models, network inference is considered a possible solution to quantify the complex relationships between heterogeneous data categories. In particular, a mutual information network is designed to explore the paths linking patient-associated variables to endpoint events, to reveal prognostic factors and to identify the best possible predictors of death. Data from a prospective, multicentre, observational study are available from a previous study, based on 4313 patients (2532 men; 64±11 years) with known (*n*=1547) or suspected (*n*=2766) CAD, who underwent high-dose dipyridamole (0.84 mg kg^−1^ over 6 min) stress echocardiography with coronary flow reserve (CFR) evaluation of left anterior descending (LAD) artery by Doppler. The overall mortality was the only endpoint analysed by Cox models. The estimated connectivity between clinical variables assigns a complementary value to the proposed network approach in relation to the established Cox model, for instance revealing connectivity paths. Depending on the use of multiple metrics, the constraints of regression analysis in measuring the association strength among clinical variables can be relaxed, and identification of communities and prognostic paths can be provided. On the basis of evidence from various model comparisons, we show in this CAD study that there may be characteristic factors involved in prognostic stratification whose complexity suggests an exploration beyond the analysis provided by the still fundamental Cox approach.

## 2. Background

In order to assess the risk of developing cardiovascular diseases, computational approaches are routinely applied and validated on several patient cohorts, providing a variety of predictive models. Cox models [1] represent the standard methodology used to estimate the effects of tests or treatments on patients' survival, after adjustment for explanatory variables and given a set of prognostic factors. Cox models present as the dependent variable a given hazard function measured at a certain time, or equivalently the probability that an individual will experience an event, such as death, within a time interval. Because the magnitude of the coefficient in correspondence to each explanatory variable would imply higher/lower associated risk, and therefore a differentiated prognosis, the complexity underlying the interdependent patterns between the variables establishes an approximate bound for their predictive power, thus justifying the search for data-adaptive rather than exact solutions.

As a measure of dependence among random variables, mutual information (MI) [2] is simple and effective, thus universally accepted. While Spearman's correlation measures a monotonic relationship (and Pearson's correlation measures a linear relationship), MI can measure non-monotonic and nonlinear relationships. That makes MI a preferred choice in most network reverse engineering approaches, and corresponding software tools. Being only based on joint and marginal data distributions, MI quantifies the dependence between two discrete variables non-parametrically, i.e. without assuming a specified distribution of the variables, and differentiating between their linear/nonlinear relationships. MI meets the zero bound under statistical independence, otherwise, its value increases when the assumption is relaxed. In general, the complexity characterizing the interactions among clinical variables is difficult to decipher, and can be managed in different ways depending on the computational model of choice. In the presence of heterogeneous clinical variables, MI allows for a quantification of their distances (in a range 0–1) within the reference space. In such regards, MI-based approaches diverge from Cox models, and in particular: (i) there is no need of optimal statistical conditions for achieving consistency of estimates, which translates into significance of the estimated coefficients; and (ii) there is no risk of multi-collinear relationships, which are detrimental to a correct interpretation of findings. As both correlative and causal forms of dependence in data can be considered valuable information, wide-spectrum computational inference is needed to assess criteria of: (i) significance, like in Cox or regression models, indicating whether an association exists and can be estimated by a coefficient; and (ii) connectivity, like in networks, indicating that some paths can be detected by measuring the strength of links between nodes. In short, networks provide data summaries at various scales, from minimally local (single node) to global (entire configuration), across several modules or communities, which are densely associated subnetworks assembled by interconnected elements [3–7].

We aim to show the relevance of both criteria, and correspondingly both approaches, with reference to a sequence of studies [8,9] involving a prognostic dataset of 4313 patients (2532 men; 64±11 years) with known or suspected coronary artery disease (CAD), analysed with stress echocardiography with wall motion and coronary flow reserve (CFR) assessment of left anterior descending (LAD) by transthoracic Doppler ultrasound, followed up for a median of 19 months, and considering mortality as the only endpoint. The data were obtained from a prospective, multicentre, observational study based on patients with known (*n*=1547) or suspected (*n*=2766) CAD, and undergoing high-dose dipyridamole (0.84 mg kg^−1^ over 6 min). Clinical characteristics are summarized in table 1 [8], and concerning the stress echocardiographic, no particular complications were reported.

**Table 1.** Clinical variables listed by patient. (Full name of the variables involved in the study, then binarized in order to compute the MI. The third column introduces the number of positives (1), whereas the fourth column details the criteria used to define a positive.)

variable ID	variable full name	no. positives	definition of a positive value
age	age	2301	age ≥ median age (66)
sex	gender	2532	male
Prev_IM	prior myocardial infarction	1114	yes
Prev_rev	prior coronary revascularization	1234	yes
Prev_CABG	prior coronary artery bypass grafting	270	yes
Prev_PCI	prior percutaneous coronary intervention	990	yes
Known_CAD	known coronary artery disease (the rest, suspected)	1547	yes
LBBB	left bundle branch block	310	yes
family	family history of CAD	1108	yes
diabetes	diabetes	979	yes
hypert	arterial hypertension	2891	yes
hyperchol	hypercholesterolemia	2364	yes
smoke	smoking habit	1311	yes
therapy	presence or absence of cardioactive medical therapy at stress test time	1798	yes
BB	beta blockers	1353	yes
CCB	calcium antagonists (calcium channel blockers)	755	yes
nitrates	nitrates	465	yes
LM	left main disease	55	≥50
RWMA	resting wall motion abnormality	1379	yes
CFR	coronary flow reserve	1421	≤2.0
N.vessels	number of diseased vessels at angiography	1092	no. vessels = 1, 2 or 3
Rest_vel_LAD	resting velocity on LAD (in cm s^−1^)	2212	≥30
Isch_SE	ischemia owing to stress echo test	765	yes
Rest_WMSI	global score of regional function of left ventricle	461	≥1.6
Peak_WMSI	score of regional kinetics at speak stress	526	≥1.6
Delta_WMSI	variation between basal and stress	108	≥0.4
ECG_SE	presence or absence of ECG changes during stress echo test	613	yes
Angor_SE	presence of angina during stress echo test	448	yes
death	death	146	yes

In short, mean CFR on LAD was 2.35±0.68; 1419 (33%) subjects had CFR≤2 and 2894 (67%) had CFR>2; stress echocardiography was positive for ischemia in 765 (18%) individuals; ischemia was assessed in 516 patients with CFR≤2 and in 249 patients with CFR>2 (36% versus 9%; *p*<0.0001).

## 3. Methods

The original analysis, following stress echo methodology, was carried by Cox survival models according to specified selection criteria for the clinical variables. The network analysis here proposed is aimed to complement the previous analyses on the data for which permission of use was allowed.

### 3.1 Cox models

Cox model computations are available in several software tools, including the R environment from which we used the ‘survival’ package [10,11] with the goal of obtaining hazard ratios (HRs), and associated confidence intervals. Univariate and multivariate models have been tested, and different algorithms have been employed for the latter category. Comparative evaluations are presented in order to rank the variables under different model types.

### 3.2 Network structure

We generated networks containing a number of nodes and edges based on the variables under examination. The initial variables that we considered in the network were those selected as significant in the reference study [8], thus forming a configuration with 17 nodes and 22 edges. The entire set of given variables was then considered in the network, forming this time an extended configuration with 29 nodes and 36 edges. The rationale for this translated analysis is that in one case the significance of variables established by a model determines the network configuration, whereas in the other case, the connectivity between all variables is taken into consideration, once MI estimates are obtained.

**Figure 1.**
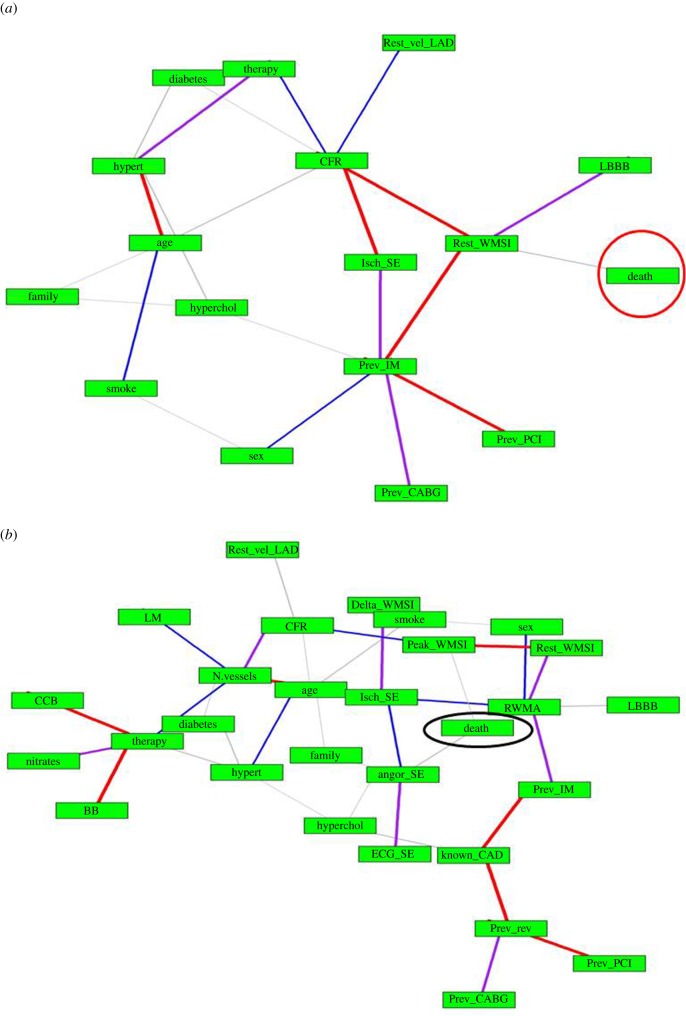
MIN. (*a*) MIN with 16 variables considered significant by Cortigiani *et al.* [8], (*b*) extended MIN with 28 variables involved in CAD.

The two configurations are shown in figure 1. The width of the edges is related to the strength of the association (MI value or edge weight). A quick visualization of associations of various types has been made available by edge colours to distinguish the association strength between variables: red edges denote associations in the top 20% of higher MI; purple edges denote the second strongest group (60–80% bin); blue edges denote the third strongest group (40–60% bin); and grey edges correspond to the still significant but least strong associations (less than 40%).

### 3.3 Network construction

The networks were assembled with every edge representing a statistically significant association measured by MI. In particular, two estimation methods were employed: the MM estimator [12] and the ARACNE algorithm [13], implemented in the ‘minet’ R package [14]. The MM refers to the Miller–Madow asymptotic bias-corrected empirical estimator, which computes the entropy of the empirical probability distribution and generates a matrix of MI values between each pair of variables. The *p*-values for the MI were computed following a Monte Carlo procedure based on 5000 random permutations for each pair of variables [15].

ARACNE stands for ‘algorithm for the reconstruction of accurate cellular networks’, and starts by assigning to each edge a weight equal to the MI between the nodes (variables), then removes the weakest of the edges forming each triangle depending on a certain threshold condition, say *T*. For instance, a useful threshold imposes removal of the edge with the weakest MI. In general, it is recommended to remove an edge if the difference between the two smallest MI in a triangle is above a certain *p*-value. In our study, we have used as our *T* a standard 0.05 *p*-value, followed by a Bonferroni correction for the number of tests. Additionally, all edges with an MI below another threshold, say *D*, can also be removed, (we fixed *D* to zero, in our application). Because the outcomes can be affected by data normalization, we used a non-normalized vector based on the inverse of the MI as the distance vector between the nodes of the network.

### 3.4 Network analysis

The mutual information network (MIN) was applied to a sample of patients with known or suspected CAD. Indications for stress echocardiography were associated with suspected CAD in 2766 (64%) subjects, and risk stratification of known CAD (i.e. history of myocardial infarction, coronary revascularization and/or angiographic evidence of >50% diameter coronary stenosis) involved the remaining 1547 (36%). Patients' characteristics are reported in table 1. Overall, 16 clinical variables in the first network, and 28 clinical variables in the second network were considered, once binarized to allow their inclusion. The ‘igraph’ R package [16] was used for network computations. The MIN was plotted as an undirected and weighted graph, with MI values as edge weights. For shortest path computations (the *shortest.paths* function was used with the Dijkstra's algorithm option), the distance between two nodes was defined as the MI inverse. The Kamada–Kawai layout was chosen to plot this network.

In order to determine the relative importance of the variables in the network, three different metrics were computed: (i) degree; (ii) betweenness centrality (BC); and (iii) Google's PageRank Centrality (PRC). The degree measures the number of edges adjacent to a node (number of significant associations of a variable). The BC measures the number of shortest paths between any pair of nodes that crosses through a given node (the number of associations between any pair of variables that involve an association with the variable under scrutiny). The PRC weights the importance of a node by assigning a higher value if the node links to other nodes which are also central to the network.

## 4. Results

### 4.1 Evidence from 16-variable mutual information network

We report in table 2 a comparison between ‘Death’ and every other variable, using the HR score, the MI and the distance in the MIN. Basically, high values for HR and MI, as well as a short path distance, identify good statistical association (i.e. association meaning that for MI and HR, we observe only if they rank the variables in a similar way or not). All three methods agree that Rest_WMSI, CFR, LBBB and Isch_SE, are the best predictors of Death. The MIN also suggests a role for Prev_IM and Prev_PCI which are not present in the two other methods. Regarding the worse predictors, all three methods agree on Hyperchol, Hypert and Smoke, whereas HR and MIN agree on Family History, and only the MIN introduces Diabetes, which is considered acceptable for the other two. Note that all the variables with a *p*-value below the significance threshold for HR are also detected by MI (Rest_WMSI, CFR, LBBB, Isch_SE, Sex, Age and Diabetes). In addition, MI shows other variables with a low *p*-value (Rest_vel_LAD and Family History).

**Table 2.** HRs (multivariate Cox) versus MIs. (Sixteen variables scenario. Shaded regions indicate not significant evidence.)

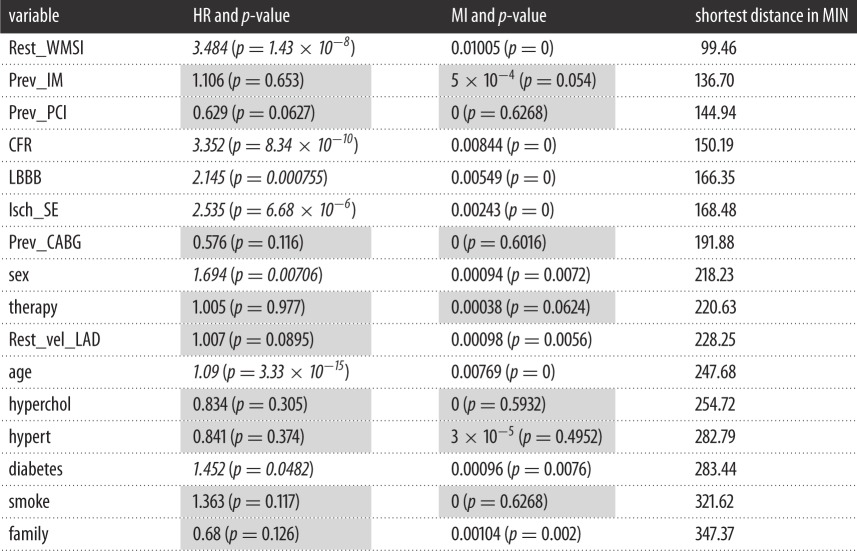

The central nodes are the most important nodes to explain the network as a whole, even if they are not necessarily the best predictors. We obtained measurements according to the three selected metrics, degree, BC and PRC, for all MIN nodes. In terms of degree, CFR and Prev_IM are the most important nodes of the network, followed by Rest_WMSI, Age and hypertension. In terms of BC, CFR is the most important variable, followed by Prev_IM, Rest_WMSI, Age and Isch_SE. In terms of PRC, CFR is again the most important node, followed by Prev_IM, Hyperchol and Rest_WMSI. All metrics agree that CFR and Prev_IM are the most central nodes to this work.

**Figure 2.**
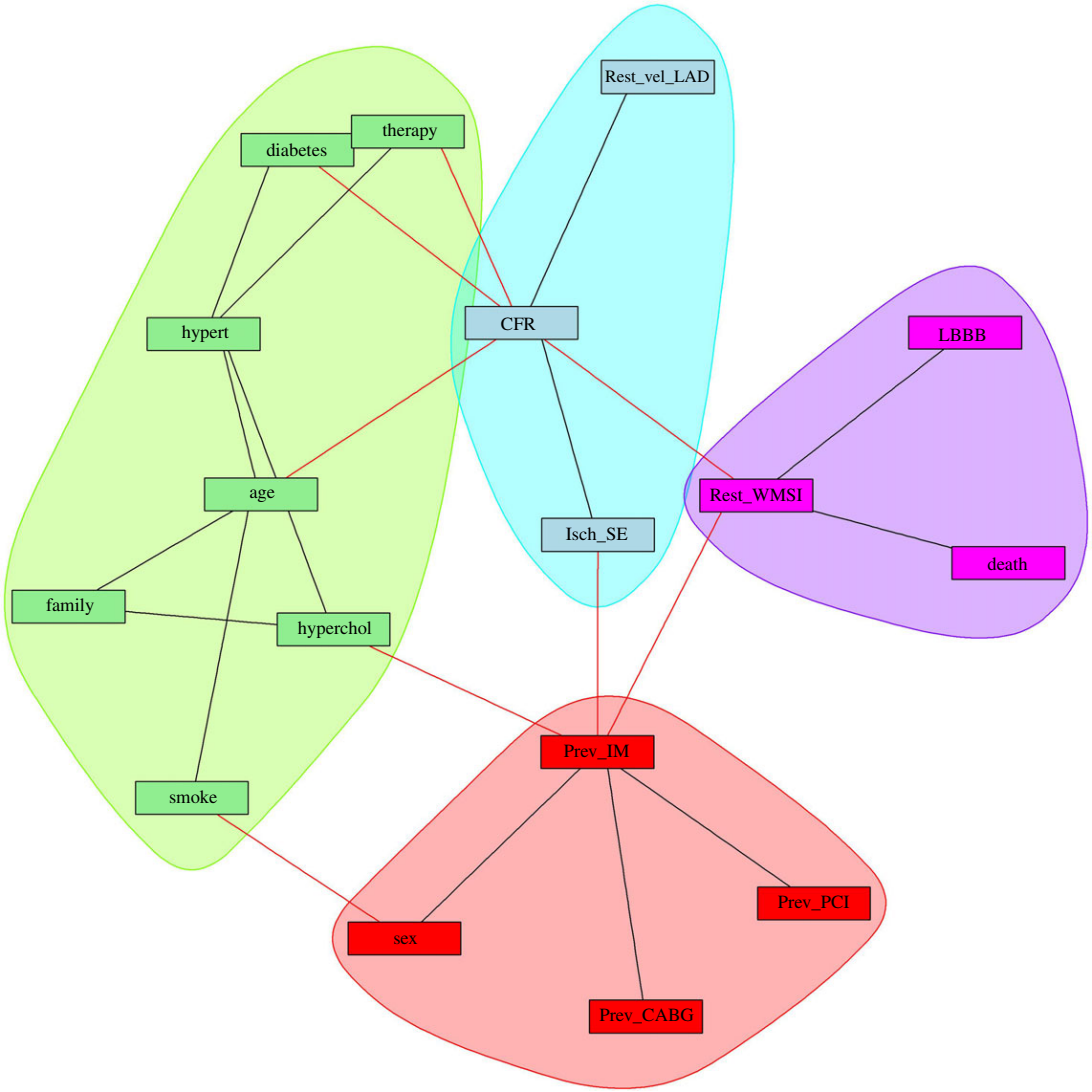
Modular map of the MIN with 16 variables.

**Figure 3.**
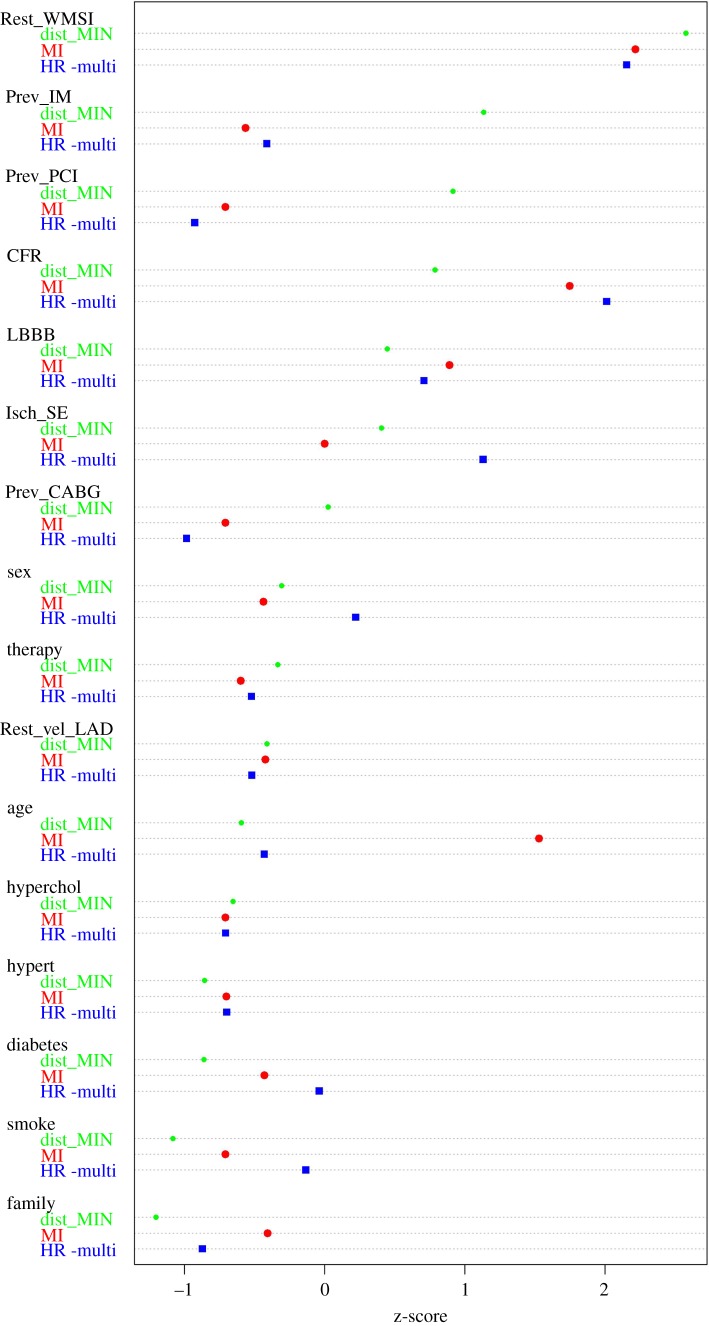
Inter-approach comparison using the MIN with 16 variables. HR_multi corresponds to the hazard ratio for the multivariate Cox model including all 16 variables. MI corresponds to the MM estimator. dist_MIN corresponds to the shortest distance between the node and Death, with distance defined as the sum of the inverse of the MI between each pair of nodes.

In general, modules include nodes which are significantly interconnected, i.e. their association strength is higher than that measured with external nodes or simply due by chance, and thus can establish functional associations among variables, providing testable hypotheses to be subjected to validation (in silico, experimental, clinical). Exploration of modularity was performed according to the Infomap method [17]. The MIN is partitioned in four modules, as it can be seen in figure 2. Red edges represent intercommunity links, whereas black edges represent intracommunity links. The purple module groups the variables most closely related to Death. The second module of interest is the green module. Here, we can find tightly related disease conditions such as hypertension, hypercholesterolemia and diabetes, together with age and smoking habits. The red module, on the other hand, seems to group all the collected previous information of the patient. For a fair quantitative comparison, we computed the *z*-score of the HR, MI and MIN-distance for all the variables and represent them in the dot chart of figure 3.

### 4.2 Evidence from 28-variable mutual information network

We report in table 3 a comparison between Death and every other variable in terms of association strength, using the HR score, the MI score and the shortest-path distance in the MIN.

**Table 3.** HRs (multivariate Cox) versus MIs. (Twenty eight variables scenario. Shaded regions indicate not significant evidence.)

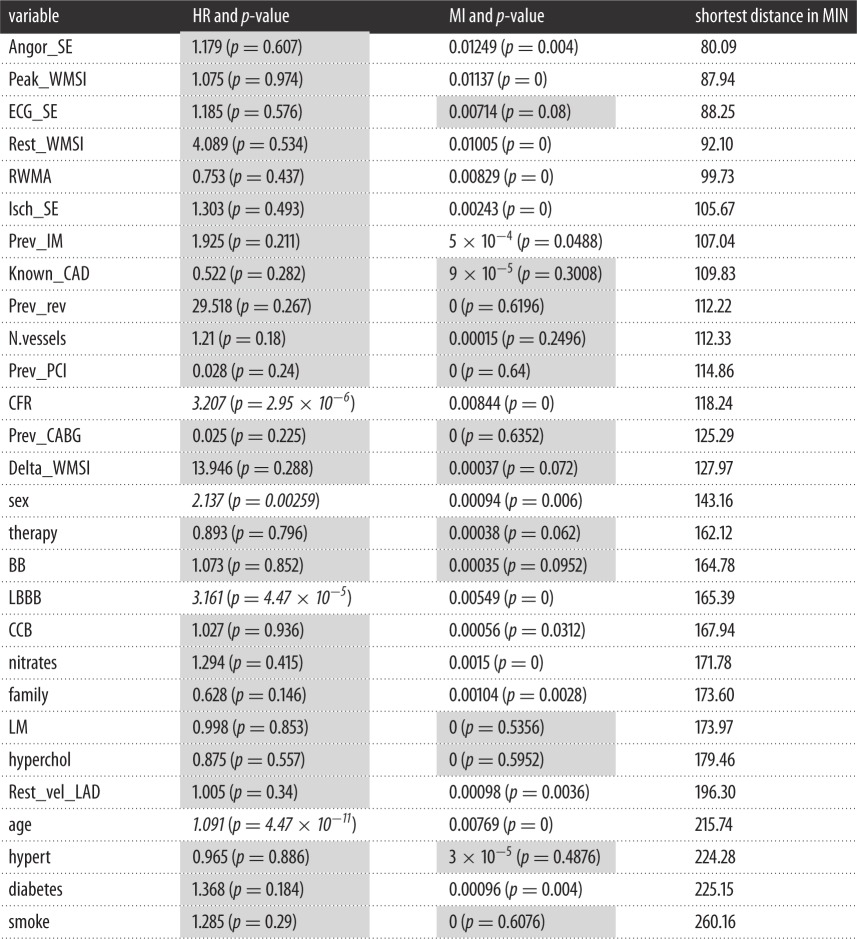

All three methods agree placing variables such as Smoke, Hypert and Hyperchol at the bottom of the rank (least prognostic value). The two MI-based methods agree regarding Angor_SE, Peak_WMI and Rest_WMI as the best predictors, whereas HR suggests CFR and LBBB. The associations with prognostic value are revealed by path analysis, showing that the best predictor of Death is Angor_SE (MI=0.012). We therefore furthermore explored the issue. In order to analyse the association between distant variables, we find their minimum distance in the network, i.e. the shortest prognostic path (see Methods). For example, the five best predictors for Death, in terms of MI, are Angor_SE (0.012), Peak_WMSI (0.011), Rest_WMSI (0.010), CFR (0.008) and RWMA (0.008). In the MIN, it can be observed that the two best predictors (Angor_SE and Peak_WMSI) are Death's neighbours, whereas Rest_WMSI and RWMA are part of the same prognostic path (note that ARACNE has removed the indirect connections, and the connections in the path have been considered as the direct association). A second path links Death to CFR through Peak_WMSI.

In terms of degree, Angor_SE, RWMA, Therapy and N.vessels are the most important nodes of the network. In terms of BC, RWMA is the most important variable, followed by Isch_SE, N.vessels and Prev_IM. For PRC, Angor_SE is again the most important node, followed by Hypert, Hyperchol, Age and N.vessels. The results indicate that Angor_SE is an important variable due to the fact that is highly associated with a greater number of variables (Death, Ischemia, Hypercholesterolemia, Family History and ECG_SE), and because is closely associated with other central variables (such as Ischemia). From a different point of view, RWMA and Ischemia are important for being crossed by paths to Death; in other words, the apparent relationship of many variables to Death is due to a relationship to RWMA or Ischemia, which are related to Death. The variables CFR and Prev_IM are less important in this extended network than in the previous one, but Prev_IM is still the fourth most important variable regarding BC, after RWMA, Ischemia and N.vessels.

**Figure 4.**
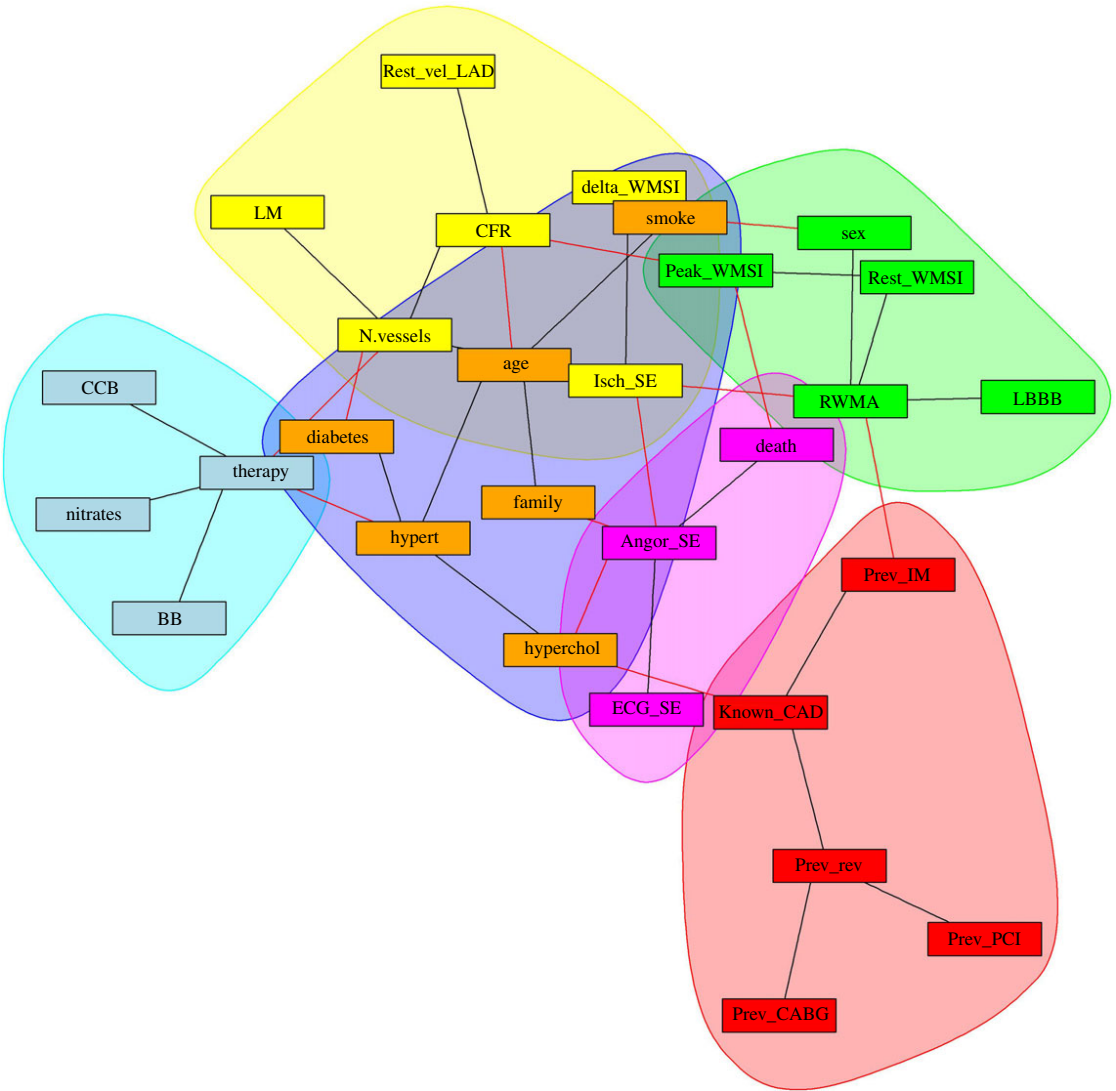
Modular map of the MIN with 28 variables.

**Figure 5.**
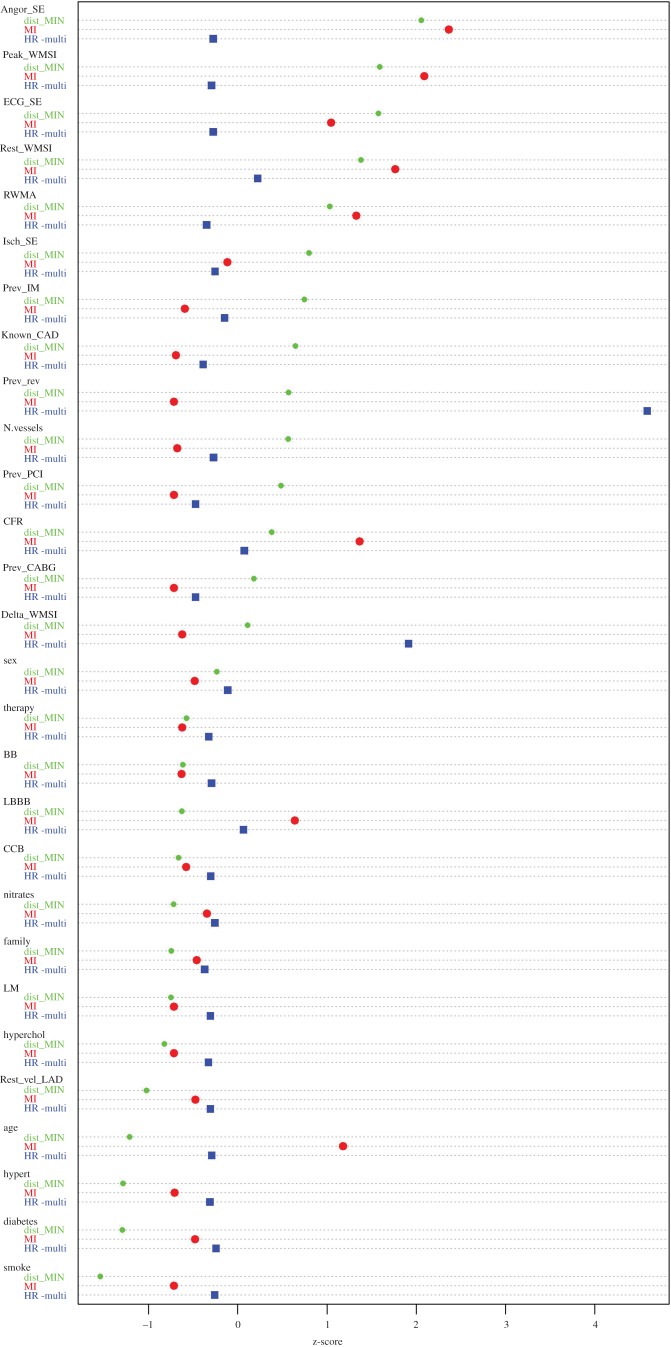
Inter-approach comparison using the MIN with 28 variables. HR_multi corresponds to the hazard ratio for the multivariate Cox model including all 16 variables. MI corresponds to the MM estimator. dist_MIN corresponds to the shortest distance between the node and Death, with distance defined as the sum of the inverse of the MI between each pair of nodes.

The MIN is partitioned in six modules, as can be seen in figure 4. The pink module groups the variables most closely related to Death. The second module of interest is the blue module, where we find hypertension, hypercholesterolemia and diabetes, together with age and smoking habits. The red module, again, seems to group all the collected previous information of the patient. The light blue module shows all therapies, which appear far from the main prognostic paths. All these variables have predictive power and they are very tightly associated among them, but their prognostic paths are mediated by the above-mentioned variables, closely associated with the output. For a more quantitative comparison, we computed the *z*-score of the HR, MI and MIN-distance for all the variables and represent them in the dot chart of figure 5.

## 5. Discussion

Cox models work by selecting a number of variables as significant, and by discarding the rest. This is done in the spirit of all regression models, and the same problem is thus occurring: when the complexity of the relationships between variables is unknown, it is hard to discriminate between variables in terms of predictive power without considering ways to regularize the model (i.e. explicitly discounting the complexity).

While regularization techniques deserve attention, we consider how to complement Cox analysis with network inference. Initially, we showed a configuration obtained with a restricted set of variables, i.e. those identified by the Cox analysis conducted by Cortigiani *et al.* [8]. Then, we considered the entire set of given clinical variables, and found a different configuration with some interesting elements. In particular, the MIN approach highlighted the role of angina during dipyridamole stress echo in Death prediction.

Furthermore, variables were organized within the network, pointing out different types of relationships: (i) classification: this is the case observed with therapies; (ii) redundancy: for instance, between Delta_WMSI and Ischemia, as the former is an indicator of the severity, which is an extension of the latter; (iii) prognostic: for the paths conducing to Death, the shortest paths means a higher association (for example, Peak_WMSI, Rest_WMSI and RWMA, in Death prediction); and (iv) modularity: specific conditions emerge through modules, for instance grouping hypertension, hypercholesterolemia, diabetes and smoking habits. These are examples of associations and information not available in traditional Cox methods, and instead naturally delivered by network approaches.

Overall, Cox analysis makes available only selectively the dependence between variables, and under specific model assumptions, whereas the examination of connectivity patterns through MI allows us to extend the estimates to all possible relationships between variables. Because in Cox models the presence of collinearity may influence the significance for some of the variables, networks suggest potential associations otherwise left unexplored. We performed extensive comparison between the identified prognostic variables for the MIN prognostic paths, the pairwise MI score among the variables, and the HRs. Some variation between the methods appeared related to the most relevant variables. Pairwise MI and the distance in the MIN show a better agreement, by construction. In the case of Death, previous work with Cox analyses and the same dataset of 4313 patients has postulated that Death is best predicted by Rest_WMSI, CFR and Ischemia.

The MIN approach suggests as a testable hypothesis that the angina during stress echocardiography plays a very important role which remained undetected from the other analysis because discarded as not significant. Then, MIN also places Rest_WMSI and CFR in the context of two prognostic paths leading to Death. In both approaches the evidence is justified, as in Cox models, the final selection excludes angina based on significance, and in MIN, its relevance depends on the estimated MI value and the computed distance from the endpoint. While this differential analysis offers elements of discussion, from a clinical point of view and in terms of model assessment, we show that networks put forth the surprising clinical dividends offered by observational evidence, namely notable symptoms, in agreement with previous evidence showing that chest pain during dipyridamole stress is a strong predictor of subsequent cardiac events, including death, with additive prognostic value over stress-induced wall motion abnormalities [18,19].

The MIN approach has strengths (for example, it can work with any type and number of variables) and limitations (we choose one of the many possible inference procedures in order to assess the connectivity among the included variables, but other procedures could be available too, and we also know that MI can be computed in different ways). In addition, our model frame is reproducible in the chosen prognostic context, i.e. CAD, and generalizable to other contexts in which similar aims are pursued. We benchmarked our proposed model to Cox models, knowing the approaches are inherently different. The principal consideration is the following: can we trust evidence of association between variables under non-optimal hypotheses? In Cox modelling, a selection for significance of associations is performed, but the price we seem to pay is that some interesting associations can be missed. In networks, we use connectivity patterns to recover some of these associations, in support of Cox evidences. Therefore, depending on the datasets which is variable, we can verify whether some forms of dependence imposed by the model of choice determine a level of detection to be considered computationally coherent (i.e. comparable across methods) and clinically relevant.

In conclusion, the recognized low diagnostic specificity and sensitivity of anginal pain during stress [20] also suggests that clinical integration is mandatory prior to any patient-oriented use of this analysis. The present data highlight the different, potentially complementary perspectives offered by the two approaches, but also the ontological difference between statistical and clinical significance. The former type of significance requires consistent estimation form the model, i.e. as the sample size increases, the estimator converges to the true value of any parameter being estimated. Instead, to achieve clinical significance, a variable must fit into the existing pathophysiological and clinical framework in order to be readily used by the clinician. Yet, the main message of the present work is that there may be more intricate relationships in prognostic stratification than those contemplated in the time-honoured and still fundamental Cox approach, and they call for supplemental investigations.

## Supplementary Material

Supplementary material

## Data accessibility

We refer the reader to published work [8] for all the data details, and linked to . We also refer the reader to the electronic supplementary material file.
